# Gene expression profiling spares early breast cancer patients from adjuvant therapy: derived and validated in two population-based cohorts

**DOI:** 10.1186/bcr1325

**Published:** 2005-10-03

**Authors:** Yudi Pawitan, Judith Bjöhle, Lukas Amler, Anna-Lena Borg, Suzanne Egyhazi, Per Hall, Xia Han, Lars Holmberg, Fei Huang, Sigrid Klaar, Edison T Liu, Lance Miller, Hans Nordgren, Alexander Ploner, Kerstin Sandelin, Peter M Shaw, Johanna Smeds, Lambert Skoog, Sara Wedrén, Jonas Bergh

**Affiliations:** 1 Department of Medical Epidemiology and Biostatistics, Karolinska Institutet, Stockholm, Sweden; 2 Department of Oncology and Pathology, Radiumhemmet, Karolinska Institutet and University Hospital, Stockholm, Sweden; 3 Genentech, San Francisco, California, USA; 4 Bristol-Myers Squibb, Princeton, New Jersey, USA; 5 Regional Oncological Center, Uppsala University Hospital, Uppsala, Sweden; 6 Genome Institute of Singapore, Singapore; 7 Department of Pathology, Uppsala University Hospital, Uppsala, Sweden; 8 Department of Surgery Sciences, Karolinska Institutet and Hospital, Stockholm, Sweden

## Abstract

**Introduction:**

Adjuvant breast cancer therapy significantly improves survival, but overtreatment and undertreatment are major problems. Breast cancer expression profiling has so far mainly been used to identify women with a poor prognosis as candidates for adjuvant therapy but without demonstrated value for therapy prediction.

**Methods:**

We obtained the gene expression profiles of 159 population-derived breast cancer patients, and used hierarchical clustering to identify the signature associated with prognosis and impact of adjuvant therapies, defined as distant metastasis or death within 5 years. Independent datasets of 76 treated population-derived Swedish patients, 135 untreated population-derived Swedish patients and 78 Dutch patients were used for validation. The inclusion and exclusion criteria for the studies of population-derived Swedish patients were defined.

**Results:**

Among the 159 patients, a subset of 64 genes was found to give an optimal separation of patients with good and poor outcomes. Hierarchical clustering revealed three subgroups: patients who did well with therapy, patients who did well without therapy, and patients that failed to benefit from given therapy. The expression profile gave significantly better prognostication (odds ratio, 4.19; *P *= 0.007) (breast cancer end-points odds ratio, 10.64) compared with the Elston–Ellis histological grading (odds ratio of grade 2 vs 1 and grade 3 vs 1, 2.81 and 3.32 respectively; *P *= 0.24 and 0.16), tumor stage (odds ratio of stage 2 vs 1 and stage 3 vs 1, 1.11 and 1.28; *P *= 0.83 and 0.68) and age (odds ratio, 0.11; *P *= 0.55). The risk groups were consistent and validated in the independent Swedish and Dutch data sets used with 211 and 78 patients, respectively.

**Conclusion:**

We have identified discriminatory gene expression signatures working both on untreated and systematically treated primary breast cancer patients with the potential to spare them from adjuvant therapy.

## Introduction

Adjuvant systemic therapy saves a significant number of lives [1-3], but many patients are subjected to unnecessary adjuvant therapies with the potential of causing more harm than good [4]. Approximately 25% [5] of all women diagnosed with breast cancer die from their disease despite having been treated according to state-of-the-art clinical guidelines [6,7]. The present lack of criteria to help individualize breast cancer treatment indicates a need for a novel technology to develop better prognostication and therapy prediction.

The stage, the tumor size and the histological grade are accepted as prognostic markers for breast cancer [8]. Estrogen receptor status, sometimes accompanied by progesterone receptor status, is the only globally accepted treatment predictive factor for hormonal therapy for primary breast cancer [6]. However, about one-half of the patients with estrogen-receptor-positive cancer fail on tamoxifen [9,10].

The microarray technology can simultaneously characterize the RNA expression profile of thousands of genes in a single tumor. Most microarray studies so far reported have utilized highly selected patient populations [11-13] and hereditary breast cancer [14], and few studies have focused on treatment prediction [15]. Prognostication of distant metastases [16,17] could potentially serve as the basis of patient selection for adjuvant therapy. There was no guarantee that the high-risk patients selected for therapy would actually benefit from it, however, and none of these previous studies addressed the important problem that a subgroup of women failed to respond to therapy.

The aim of our project was to use gene expression profiling to identify patients whose tumors have a low malignant potential, making adjuvant therapy unnecessary and potentially harmful, and to identify patients in need of more effective adjuvant therapies. Furthermore, we wanted to show that the expression profile worked irrespective of primary adjuvant therapy or not and provided independent information to the established clinical factors.

## Materials and methods

### Study population

**Figure 1:**
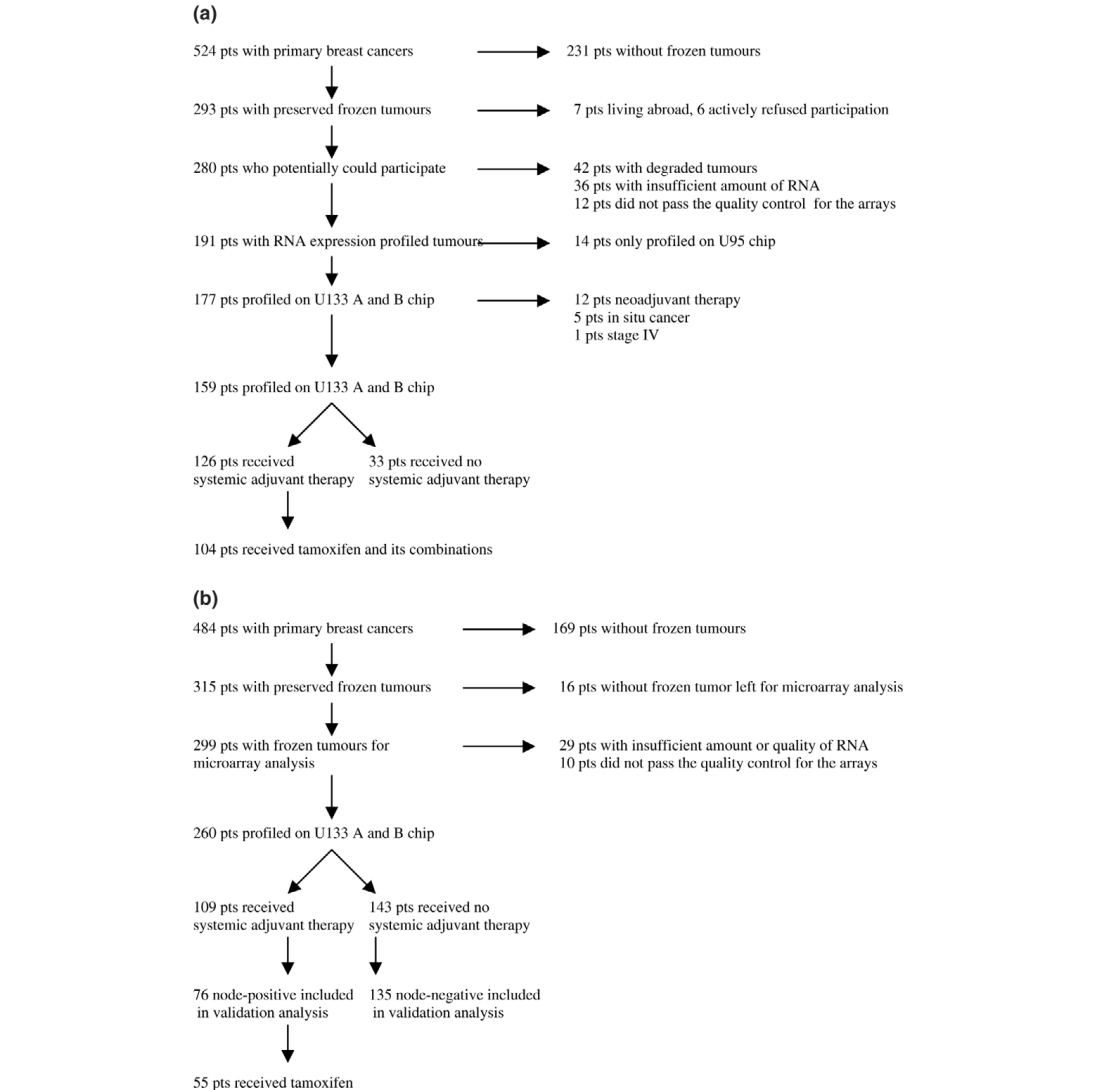
Description of exclusion criteria for all patients (pts) operated on for primary breast cancer at **(a) **Karolinska Hospital, 1994–1996 and **(b) **Uppsala University Hospital, 1987–1989.

We included all breast cancer patients that were operated on at the Karolinska Hospital from 1 January 1994 to 31 December 1996 (*n *= 524), identified from the population-based Stockholm–Gotland breast cancer registry established in 1976. Available tumor material was frozen on dry ice or in liquid nitrogen and was stored in -70°C freezers. Figure 1 shows the details of various exclusions leading to the final 159 patients for analysis. The ethical committee at the Karolinska Hospital approved this microarray expression project.

The different reasons for exclusion were not influenced by age at diagnosis (Table 1). The 231 tumors that were not analyzed using expression profiling had a lower mean diameter, had fewer mean affected lymph nodes, and had fewer individuals with recurrent disease at the end of the study period (Table 1). For those excluded for other reasons, there did not seem to be a selection based on age or stage of the disease, compared with those patients included in the study (Table 1).

The Stockholm–Gotland Breast Cancer Registry, supplemented with patient records, were examined for information on the tumor size, the number of retrieved and metastatic axillary lymph nodes, the hormonal receptor status, distant metastases, the site and date of relapse, initial therapy, therapy for possible recurrences, the date and cause of death. Tumor sections from the primary tumors from patients with array profiles were classified using Elston–Ellis grading [18] by a blinded pathologist (HN).

In the adjuvant setting tamoxifen and/or goserelin is normally used for hormonal treatment, but mostly intravenous cyclophosphamide, methotrexate and 5-fluorouracil (CMF) on days 1 and 8 was used as adjuvant chemotherapy, except in high-risk patients who were offered inclusion in the Scandinavian Breast Group 9401 study [19]. After primary therapy, patients were recommended to have regular clinical examinations and yearly mammograms, in addition to laboratory and X-ray tests guided by clinical signs and symptoms. Patients were normally followed for 5 years. Patients followed up outside the Karolinska Hospital were tracked using a unique personal identification number. There was no loss to follow-up.

The relapse site, date of relapse, relapse therapy and date of death were ascertained in May 2002. The average follow-up was 6.1 years. Cause of death was coded as death due to breast cancer (including those with distant metastases but dying from other causes), death due to other malignancies and death due to nonmalignant disorders. Through the population-based Swedish Cancer Registry, second primary malignancies were identified.

### Validation data

For validation we used population-derived primary breast cancer patients receiving primary therapy from 1987 to 1989 in the county of Uppsala, Sweden [20-22]. From the initial set of 315 patients, representing 65% of all breast cancer patients in Uppsala county during these years, we were able to obtain quality-controlled RNA expression profiles from 260 frozen tumors (including two patients with neoadjuvant tamoxifen) (Fig. 1). A further follow-up of events was carried out with a 1999 deadline. Seventy-six lymph-node-positive patients received adjuvant, mostly intravenous, 3-weekly CMF-based therapy (premenopausal patients) or adjuvant tamoxifen (postmenopausal patients) [20]. Some node-negative patients also received adjuvant therapy as previously described [20]. One hundred and thirty-five node-negative patients did not receive adjuvant therapy. All tumors have been confirmed to have invasive cancer and have been graded according to Elston–Ellis, except for one patient with missing primary tumor slides but with the presence of axillary lymph nodes, thus confirming invasiveness. The ethical committee at the Karolinska Institutet approved this RNA expression study.

### RNA preparation

RNA extraction was performed according to the RNeasy mini protocol (Qiagen, Hilden, Germany). In brief, a portion of the deep frozen tumor was cut into minute pieces and transferred into test tubes (maximum 40 mg/tube) with RLT buffer (RNeasy lysis Buffer, Qiagen, Hilden, Germany), followed by homogenization for around 30–40 s. Proteinase K was then added and the samples were treated for 10 min at 55°C. This step was introduced during the project [23] because most initial preparations without this step resulted in either poor RNA yield and or poor RNA quality. Total RNA was then isolated using Qiagen's microspin technology. DNase was also added to some samples to further increase the RNA quality. The quality of the RNA was assessed by measuring the 28S:18S ribosomal RNA ratio using an Agilent 2100 bioanalyzer (Agilent Technologies, Rockville, MD, USA). All samples with RNA of high quality were then stored at -70°C until microarray analyses.

### Microarray profiling

Preparation of *in vitro *transcription products and oligonucleotide array hybridization and scanning were performed according to the protocol of Affymetrix (Santa Clara, CA, USA). In brief, the amount of starting total RNA for each probe preparation varied between 2 and 5 μg. The *in vitro *transcription reactions were performed in batches to generate biotinylated cRNA targets, which were subsequently chemically fragmented at 95°C for 35 min. Fragmented and biotinylated cRNA (10 μg) was hybridized at 45°C for 16 hours to Affymetrix high-density oligonucleotide array human HG-U133 set chips. The arrays were washed, and were then stained with streptavidin–phycoerythrin (final concentration, 10 μg/ml). The array was then scanned according to the manufacturer's instructions (*Affymetrix Genechip*^® ^*Technical Manual*, 2001; Affymetrix). The scanned images were inspected for the presence of obvious defects (artifacts or scratches) on the array. In the case of visible microarray artifacts, the sample was rehybridized and rescanned on new chips using the same fragmented probe. The raw expression data were normalized using the global mean method [24].

A statistical data filter was applied to reduce noise and to obtain a useful and relevant probe set to identify markers that were highly correlated to clinical parameters. The detail is provided in the Supplementary Report (Additional file 1). This led to 6573 final probe sets for analysis, consisting of 3393 probe sets from U133A and 3180 probe sets from U133B. All analyses were performed using natural-log-transformed expression values.

### Primary analysis and validation data sets

The primary statistical analysis was based on comparing good prognosis and poor prognosis, where poor prognosis was defined as distant relapse or death from any cause within 5 years of diagnosis. For comparison, a secondary analysis was performed limiting poor prognosis to distant relapse and death due to breast cancer. The secondary classification resulted in seven patients switching from the poor to good prognosis group.

In order to maximize the statistical power, we initially used the whole Stockholm cohort (*n *= 159) as the training set for choosing an optimal gene set and identifying risk groups in the hierarchical cluster analysis. This cohort was a mixture of patients without adjuvant therapy and 126 patients treated with CMF, tamoxifen, megesterolacetat, goserelin or some combinations thereof. We checked the consistency of the analysis of the whole cohort against the subset of patients treated with tamoxifen and its combinations (*n *= 104) and against all systematically untreated patients (*n *= 33).

To validate the results we used independent datasets from Uppsala, consisting of 76 node-positive adjuvant-treated patients and 135 node-negative untreated patients, and from the Dutch study of 78 untreated node-negative patients [16], referred to as the van't Veer data.

### Optimal gene selection, definition of the poor-prognosis score and statistical analysis

An optimal set of predictors was chosen using a leave-one-out cross-validation procedure performed on the training set (Additional file 1). Class prediction using *k *genes was carried out using a diagonal linear discriminant analysis method [25], which is a variant of the standard maximum-likelihood discrimination rule. The class predictor score *S *is computed from the top *k *genes. A patient with *S *> 0 is assigned to the poor-prognosis group, and otherwise to the good prognosis group. We will thus refer to *S *as the poor-prognostic score or the risk score.

To investigate whether the risk score had an independent predictive value over the standard clinical variables, the risk score *S *(high–low, with 'high' defined as *S *> 0) was included in a multivariate logistic regression analysis with 5-year status as the outcome variable. To obtain unbiased estimates, the scores for patients in the training set were computed from the leave-one-out procedure; because of dependence between samples, however, this procedure tends to produce optimistic standard errors [26]. We did not attempt to correct the standard errors, because the result was also validated in independent datasets. The clinical variables were the age at diagnosis, tumor grade, tumor size and lymph node metastasis, estrogen receptor status (positive–negative) and progesterone receptor status (positive–negative). The tumor size and lymph node metastases were entered into the model in the form of a stage variable. These clinical predictors were initially compared between the good-prognosis and poor-prognosis groups.

Unsupervised hierarchical clustering of the training data was used to identify flexible risk groups; here we used the Euclidean distance with complete linkage. For validation data, we used supervised clustering based on the assignment of samples to the cluster with the closest centroid. The standard Euclidean distance was used for Uppsala datasets, but for the van't Veer dataset, because of different scales and possible outliers, the distance was based on Spearman rank correlation.

To obtain a better description of the prognosis of the patients during the follow-up, we also performed survival analysis, enabling us to use full survival information not just the 5-year status. The Cox proportional hazard model was used to assess the additional contribution of the prognosis score after adjusting for the clinical variables.

## Results

Clinical characteristics of the patients (*n *= 159) in this study (Table 2) showed that those who died or who had distant metastases (*n *= 38) more often had tumors ≥ 21 mm in size (*P *= 0.06), had a higher mean diameter (*P *= 0.05), were more often progesterone-receptor-negative (*P *= 0.01) and less often received endocrine therapy (*P *= 0.03). No significant difference was detected in the proportion of patients receiving chemotherapy or radiotherapy. A similar pattern was observed when the analyses were limited to breast-cancer-specific deaths (Additional file 1).

Of the 159 patients in the training set, 38 patients died or relapsed by 5 years and were thus defined as the poor-prognosis group. Twenty-six of these patients had distant metastases by 5 years, and 12 patients died within 5 years without diagnosis of distant relapse; six of the 12 deaths were due to breast cancer. The remaining 121 patients were defined as the good-prognosis group. Of these patients, after more than 5 years of follow-up, four patients died without recurrence of breast cancer and four patients had distant relapse.

The leave-one-out procedure (Additional file 1) suggested *k *= 64 genes as an optimal number of genes for separating the patients with good prognosis and poor prognosis, giving an overall error rate of 33%. The list of these genes is presented in Additional file 1. Among the genes that have higher expression in tumors with good prognosis, we found cyclin-dependent kinase inhibitor 1 C (CDKN1C), spinal-cord-derived growth factor B, homeobox A5 (HOXA5) and insulin-like growth factor 1 (IGF1). Of the genes highly expressed in the poor-prognosis group we found genes primarily involved in cell-cycle regulation.

To check whether the expression profile has an independent predictive value compared with standard clinical factors, we performed a multivariate logistic regression analysis of the 5-year status. The results (Table 3) showed high risk associated with the poor-prognosis score (odds ratio, 4.19; 95% confidence interval, 1.49–11.77) after adjusting for age, stage, grade, estrogen receptor status and progesterone receptor status. Of these clinical variables, only progesterone-receptor-positive status was associated with better prognosis (odds ratio, 0.35; 95% confidence interval, 0.12–0.99). When we considered breast cancer endpoints (Additional data 1), the result for the microarray-based prognostic score is more significant than for overall endpoints (odds ratio, 10.64; 95% confidence interval, 2.91–38.87). The multivariate Cox regression analysis of the overall and breast cancer endpoints (Additional data 1) produced similar results to those of the previous logistic regression analysis.

**Figure 2:**
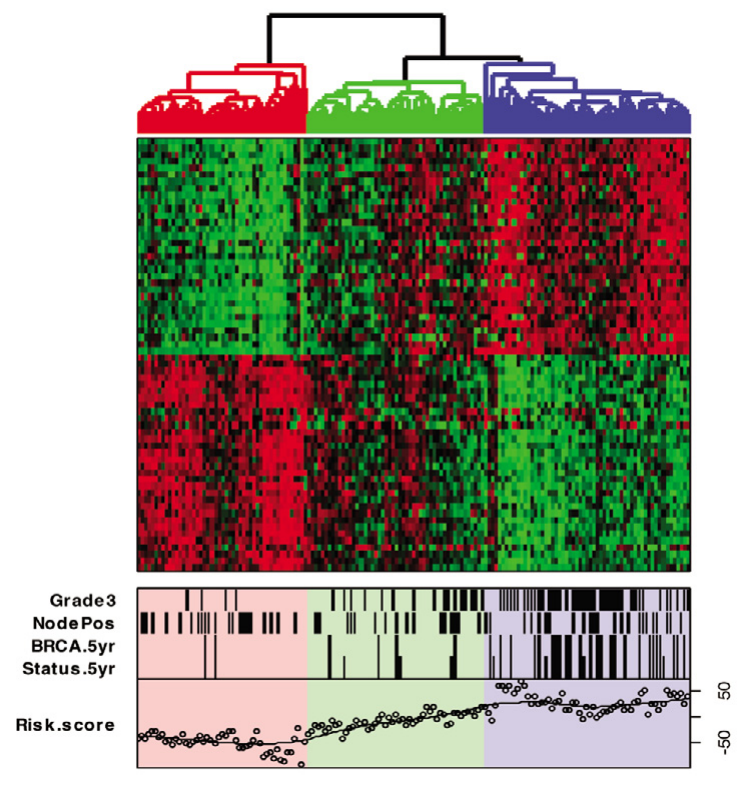
Unsupervised hierarchical clustering of the Stockholm cohort (*n *= 159) using the 64-gene set. Each column refers to a patient and each row to a gene. Red indicates a high value of gene expression, and green indicates a low value. The list of genes is presented in Additional file 1, in the same order as they appear on the plot. Risk.score, computed by linear discriminant analysis and used here only to describe the clusters. Status.5 yr, black if the corresponding patient had distant metastasis or died within 5 years. BRCA.5 yr, black if the death was due to breast cancer. NodePos, black if the corresponding patient was lymph-node-positive; Grade3, black if the patient had Elston grade 3.

**Figure 3:**
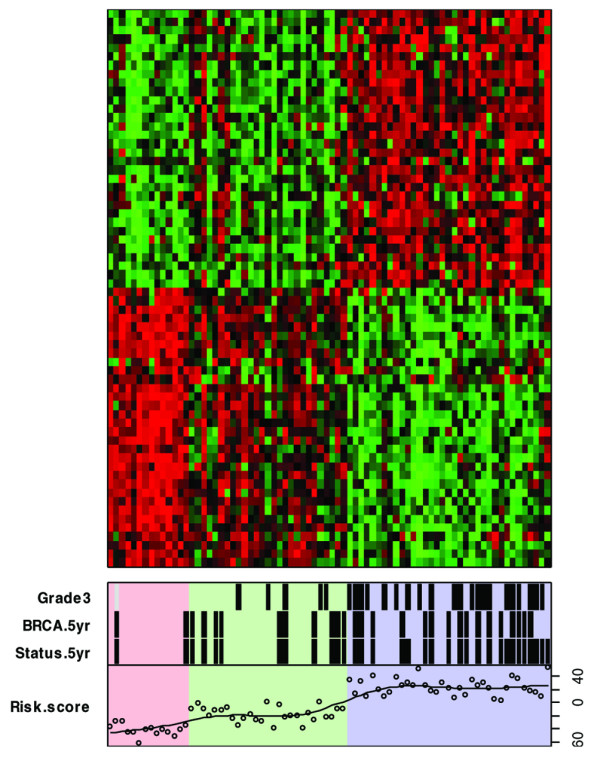
Supervised clustering of the node-positive treated cohort in Uppsala (*n *= 76) using the 64-gene set. The accompanying variables have the same meaning as in Fig. 2.

The use of the risk score as a classifier offered only a rigid classification of the patients into good-prognosis and poor-prognosis groups. To overcome this rigidity, we performed a more flexible classification by hierarchical clustering of 159 patients using the 64-gene set; here the risk score was only used for a description of the resulting clusters. The clustering procedure identified three expression-based subgroups with significantly distinct prognoses (Fig. 2), arranged from left to right in increasing risk level. There were 59 patients in the high-risk cluster, of which 29 patients (49%) had distant metastases or died within 5 years (Table 4). The subset of the patients treated with tamoxifen and its combinations (*n *= 104) revealed the high-risk signature in 33 patients, of which 16 patients (48%) had distant metastases or died within 5 years (Table 4). The high-risk profile was validated by observations from an independent group of adjuvant-treated patients from Uppsala (*n *= 76) (Fig. 3), where 21 out of 35 patients (60%) from the high-risk cluster had distant metastases or died within 5 years (Table 4). As seen in Fig. 2, the clusters were correlated with tumor grade but not with nodal status.

**Figure 4:**
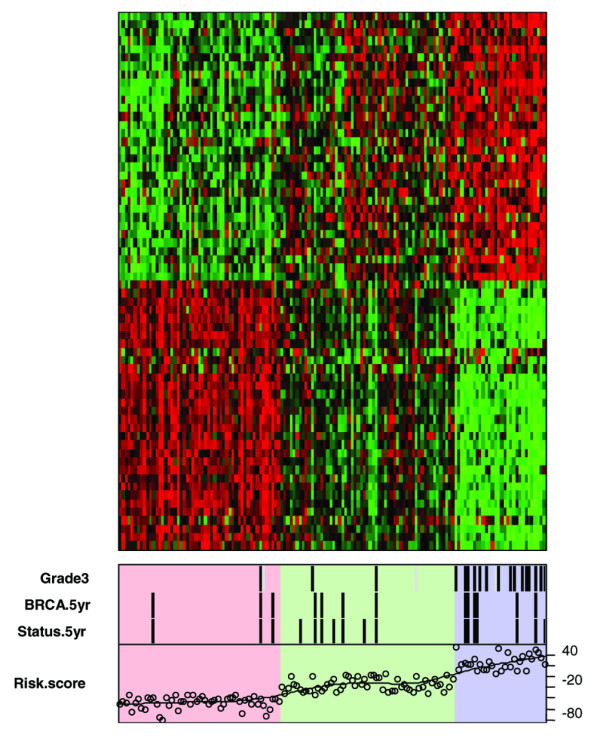
Supervised clustering of the node-negative untreated cohort in Uppsala (*n *= 135) using the 64-gene set. The accompanying variables have the same meaning as in Fig. 2.

**Figure 5:**
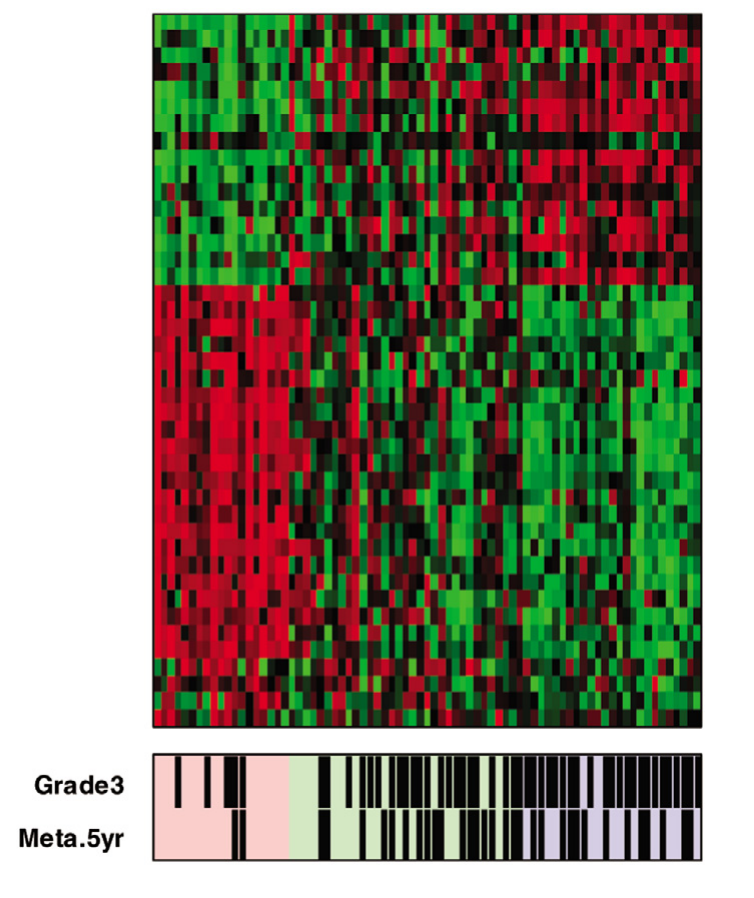
Supervised clustering of the van't Veer cohort (*n *= 78) using 42 genes of the 64-gene set. Meta.5 yr, black if the patient had distant metastasis within 5 years.

Among the untreated subgroup from Stockholm (*n *= 33), 11 out of 16 patients (69%) of the high-risk subgroup reached the primary endpoint by 5 years (Table 4). Examinations of the clustering of the untreated patients from Uppsala (*n *= 135) (Fig. 4) and from the van't Veer cohort (*n *= 78) (Fig. 5) indicated that the high-risk cluster had a consistently higher 5-year event rate than the other clusters in the same cohort (Table 4). A similar result was obtained for the van't Veer cohort when the additional 19 patients used for validation in the original publication [16] were added: 57% of the high-risk group had a 5-year event rate (data not shown).

To identify women who will do well with or without adjuvant treatment, we examined the clustering of the untreated patients in Figs 4 and 5. The rates of death or distant metastases within 5 years were three out of 53 patients (5.7%) and four out of 25 patients (16%), respectively. Among the treated groups (Figs 2 and 3), the same expression profile is associated with the lowest event rates of two out of 49 patients (4.2%) and two out of 14 patients (14%), respectively, compared with the other clusters (Table 4). In the tamoxifen-treated subgroup in Stockholm, none of the 38 patients with a low-risk profile had any event by 5 years (Table 4).

To summarize, the gene profiling revealed a statistically significant 5-year outcome result for treated patients in the Stockholm (*n *= 104, *P *< 10^-6^) and the Uppsala (*n *= 76, *P *= 0.002) cohorts, respectively (Table 4). The expression profile also provided similar 5-year outcome data for patients not receiving adjuvant therapy (Stockholm cohort, *n *= 33, *P *= 0.002; van't Veer cohort, *n *= 78, *P *= 0,01; Uppsala cohort, *n *= 135, *P *= 0.02) (Table 4).

**Figure 6:**
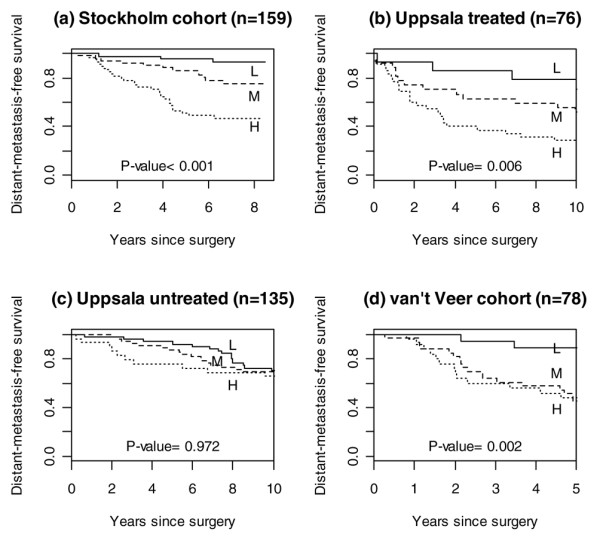
Kaplan–Meier survival curves of the risk clusters found in **(a) **the Stockholm cohort, **(b) **the Uppsala treated cohort, **(c) **the Uppsala untreated cohort and **(d) **the van't Veer cohort. L, low-risk group; M, medium-risk group; H, high-risk group. The *P *value in each plot, computed in a Cox regression, is for simultaneous comparison of all three curves for the whole follow-up period.

To gain a better description of the results throughout the follow-up period and across studies, we computed the Kaplan–Meier survival curves of the risk clusters we found in all datasets (Fig. 6). For the high-risk group in all studies, survival tended to drop fastest in the first 5 years after surgery and to level off after 5 years. This means that the 5-year survival rate provided the best comparison between risk clusters. The results were mainly consistent across studies and confirmed the expected survival patterns of risk groups (Fig. 6). For the node-negative untreated Uppsala patients (Fig. 6c), the lack of significance is due to the convergence of the survival curves at around 8 years after surgery. If we limit the comparison to 5-year survival, the survival curves are significantly different (i.e. consistent with the result in Table 4).

## Discussion

Several consensus documents [6,7] have underlined the lack of useful prognostic and predictive factors beyond tumor size, axillary lymph node status, histological grade and hormone receptor status. Our expression profile, consisting of 64 genes, was better than those in clinical use today, including the factors histological grade, tumor stage and age. Using gene expression profiling we were able to stratify patients into those that did well and where treatment did not appear to contribute, and into those with an aggressive tumor who failed to respond or developed resistance to the used adjuvant therapies.

### Analysis of adjuvant-treated and untreated groups

Our analytic approach improved the previous studies of breast cancer prognosis using microarray gene expression data by jointly analyzing adjuvant-treated patients and untreated patients. A previous Dutch study identified 70 prognostic genes from an analysis of untreated patients [16], but provided no indication of who might fail to respond to adjuvant therapy. Other studies involving treated patients also did not discuss the treatment assessment [27].

Any evaluation of adjuvant therapy must consider three types of patients: type A, those patients who do well without treatment; type B, those patients who do poorly without treatment, but may benefit from treatment; and type C, those patients who do poorly despite treatment. It is clear that type-A patients should not be treated, while type-C patients require new treatment protocols that were not available during the study periods 1987–1989 and 1994–1996, respectively. Our results indicate that the low-risk cluster consists mostly of type-A patients and the high-risk cluster consists mostly of type-C patients. The medium-risk group does not provide such clear information. In the present study we identified that around 30% of the patients in the Stockholm cohort were of low risk, hence requiring no adjuvant therapy, and that almost 40% were of high risk, for whom the existing adjuvant therapy failed. Almost three-quarters of the patients therefore did not benefit from treatment. To our knowledge, no previous study has stated this as explicitly.

One weakness of the current study as well as all of the previous studies is the inability of the gene expression profile to identify patients that will actually benefit from treatment (type-B patients). Without a randomized trial, this goal appears difficult to reach.

### Molecular biology of the markers

Twenty-two of the 64 genes have unknown function, while the other genes represent various biological pathways such as DNA replication and transcription, cell-cycle regulation, cell adhesion and metastasis (Table 5). Among the genes associated with low-risk tumors we found CDKN1C, PDGFD, IGF1, HOXA5, SLIT2 and PTN, and many of the genes associated with high-risk tumors appeared to be involved in cell-cycle regulation (including CDC2, CDC20, BUB1B, PRC1 and RRM2) and in transcription (such as TOP2A).

Some of the genes have been reported to be involved in breast cancer. CDKN1C, a tumor suppressor gene that regulates cell proliferation, was recently found to be downregulated in metastatic tumors [28]. Circulating IGF1 levels are associated with increased breast cancer risk [29]. The HOXA5 gene has been shown to play an important role in breast tumorigenesis. Its expression is higher in normal breast epithelium than in breast carcinomas, and expression of HOXA5 is lost in over 60% of breast tumors and cell lines, largely due to methylation, while its overexpression has been shown to induce apoptosis [30]. HOXA5 functions as a positive regulator of p53 transcription, and breast cancer cell lines and breast tumors display a coordinate loss of p53 and HOXA5 mRNA and protein expression [31]. Other direct targets of HOXA5 are the progesterone receptor and PTN genes. Microarray analysis has shown significantly lower expression of pleiotrophin in breast carcinomas [32,33]. In agreement with this, both HOXA5 and PTN were associated with the low-risk tumors in our data set. SLIT2 is also a potential tumor suppressor gene. Promoter methylation associated with reduced SLIT2 expression was found in 43% of breast tumors [34]. The TOP2A gene, which was associated with the high-risk tumors, was recently identified as one of the genes of a general cancer metasignature [35]. TOP2A has an essential role in DNA replication and is a molecular target for many anticancer drugs. In breast cancer, gene copy aberrations of the TOP2A gene have been detected [36].

Only three of the 64 genes in our study were among the 70 genes found to have a prognostic value identified by van't Veer and colleagues [16]: LOC51203, PRC1 and L2DTL. Table 5 compares the gene functions according to the gene ontology. To obtain an assessment of genome-wide correlation, we obtained the *t *statistics from 6434 genes in common between the Stockholm and van't Veer cohorts, and obtained a correlation of 0.31. The lack of strong correlation is probably not surprising, as there are several differences between the two studies. Firstly, the tumors analyzed came from different patient cohorts; the Stockholm and Uppsala cohorts were population-derived with clearly described inclusion and exclusion criteria (Fig. 1), while the van't Veer cohort of lymph-node-negative patients were preselected to have a distant recurrence versus no relapse within 5 years. Secondly, different gene expression platforms were used in the two studies, probably resulting in both different initial gene sets being quantified and examined, and in different relative quantification values for a given gene. Thirdly, different methodologies may have been used in tumor archiving and RNA preparation. Finally, different statistical and filtering approaches were used to obtain a subset of genes that make up the best prognostic gene sets.

## Conclusion

The adjuvant therapy experienced by the patients in this study included CMF for the premenopausal patients and tamoxifen for the postmenopausal patients. The use of anthracycline-based and taxane-based therapy and more optimal endocrine therapy strategies might alter the distribution of patients, especially moving individuals from type C to type B [37-41], as experienced with the Her-2 status and adjuvant anthracycline therapy [42]. Our results would suggest that the individuals who would potentially benefit from aggressive therapies with anthracyclines or taxanes would be primarily those identified by our classifier as the high-risk cluster, but that has to be investigated in a prospective study.

In summary, using the expression profiles of 64 genes we developed a prognostication of breast cancer patients after surgery. We identified that almost three-quarters of early breast cancer patients might not benefit from adjuvant therapy because of superior outcome or because of failing to respond to current adjuvant therapy. With recent molecular studies showing that breast cancer consists of a number of different subgroups with unique prognostic properties, the conventional management of breast cancer patients seems ripe for improvement.

## Abbreviations

CDKN1C = cyclin-dependent kinase inhibitor 1C; CMF = cyclophosphamide, methotrexate and 5-fluorouracil; HOXA5 = homeobox A5; IGF1 = insulin-like growth factor 1.

## Competing interests

Peter Shaw, Fei Huang and Xia Han are employed by the Bristol-Myers Squibb Pharmaceutical Research Institute, Princeton, NJ, USA. Lukas Amler is employed by Genentech, San Francisco, California, USA.

## Authors' contributions

YP and AP performed the biostatistical analysis and co-wrote the manuscript. JB, ALB, SE, KS, LS contributed tumors, coordinated tissue and clinical data collection and extraction of RNA for the Stockhom cohort. LA, XH, FH and PMS coordinated and performed the microarray data collection for the Stockholm cohort, and co-wrote the manuscript. PH and SW conceived and co-wrote the manuscript. LH, SK, ETL, LM, JS contributed tumors, coordinated tissue collection, and clinical and microarray data collection for the Uppsala data. HN performed the pathological assessment of the samples. JB was the principal investigator of the project and co-wrote the manuscript.

## Supplementary Material

Additional file 1 Supplementary Report presenting (i) details of gene filtering, (ii) details of cross-validation procedure to choose 64-gene signature, (iii) list of 64 genes, and (iv) other statistical analyses based on secondary endpoints. Click here for file

## Figures and Tables

**Table 1:** Characteristics of patients operated for breast cancer at the Karolinska Hospital 1994–1996

Patient categories	All patients (*n *= 524)	No available tissue^a ^(*n *= 231)	Excluded for other reasons^b ^(*n *= 134)	Included for analysis (*n *= 159)
Mean age at breast cancer diagnosis (years)	58	57	58	58
Mean tumor size (mm)	20	16	24	22
Proportion of patients with tumor size <21 mm (%)	68	77	57	62
Proportion of patients with positive lymph nodes (%)	26	16	32	38
Proportion deceased (%)	20	12	26	24

^a^No frozen tumors in the tumor bank (*n *= 231).

^b^Living abroad (*n *= 7), actively refused participation (*n *= 6), degraded tumors (*n *= 42), insufficient amount of RNA (*n *= 35), not passed the QC for the arrays (*n *= 12), profiled on the U95 chip (*n *= 14), neoadjuvant chemotherapy (*n *= 12), *in situ *cancer (*n *= 5) and stage IV at diagnosis (*n *= 1).

**Table 2:** Univariate comparison of clinical variables among patients with good prognosis and poor prognosis

	Alive (*n *= 121)	Deceased (*n *= 38)	*P *value
Proportion of patients with poor prognosis score (%)	0.34	0.74	<0.0001
Mean age at breast cancer diagnosis (years)	57.5 (±12.4)	58.8 (±16.8)	0.59
Mean tumor size (mm)	21.3 (±11.5)	25.6 (±12.6)	0.05
Proportion of patients with tumor size <21 mm (%)	0.65	0.47	0.06
Proportion of patients with positive lymph nodes (%)	0.37	0.39	0.71
Proportion of patients with grade I (%)	0.23	0.08	0.06^a^
Proportion of patients with grade II (%)	0.41	0.36	
Proportion of patients with grade III (%)	0.36	0.56	
Proportion of patients with estrogen-receptor-positive tumors (%)	0.83	0.79	0.61
Proportion of patients with progesterone-receptor-positive tumors (%)	0.77	0.55	0.01
Proportion of patients receiving chemotherapy (%)	0.18	0.21	0.69
Proportion of patients receiving endocrine therapy (%)	0.76	0.58	0.03
Proportion of patients receiving radiotherapy (%)	0.51	0.39	0.21

Continuous variables compared using *t *tests; proportions compared using chi-squared tests.

^a^Combined testing using the chi-squared test.

**Table 3:** Multivariate logistic regression of the 5-year disease free status in relation to the poor-prognosis score and other clinical variables

	All events (*n *= 159) (number of events = 38)	
	
	Odds ratio (95% confidence interval)	*P *value
Poor-prognosis score	4.19 (1.49–11.77)	0.007
Age (per 10 years)	1.11 (0.79–1.54)	0.55
Stage		
Stage 2 versus 1	1.28 (0.4–4.08)	0.68
Stage 3 versus 1	1.11 (0.42–2.95)	0.83
Elston grade		
Grade 2 versus 1	3.32 (0.63–17.56)	0.16
Grade 3 versus 1	2.81 (0.5–15.74)	0.24
Estrogen-receptor-positive	2.94 (0.76–11.28)	0.12
Progesterone-receptor-positive	0.35 (0.12–0.99)	0.05

**Table 4:** Prognosis of the clusters identified in the training and validation sets

Cohort		Risk cluster				*P *value^a^
			
		Total	Low	Medium	High	
Training set						
Stockholm	*n*	159	49	51	59	
	5-year event^b^	38 (24%)	2 (4.2%)	7 (14%)	29 (49%)	<10^-7^
Treated with adjuvant therapy						
Stockholm tamoxifen only	*N*^c^	104	38	33	33	
	5-year event	22 (21%)	0 (0%)	6 (18%)	16 (48%)	<10^-6^
Uppsala node-positive	*n*	76	14	27	35	
	5-year event	33 (43%)	2 (14%)	10 (37%)	21 (60%)	0.002
Untreated with adjuvant therapy						
Stockholm	*n*^c^	33	7	10	16	
	5-year event	11	1 (14%)	0 (0%)	11 (69%)	0.002
Uppsala node-negative	*n*	135	51	55	29	
	5-year event	17 (12%)	3 (5.8%)	7 (13%)	7 (24%)	0.02
Van't veer	*n*	78^d^	19	33	25	
	5-year event	33	2 (17%)	18 (53%)	13 (55%)	0.01

^a^*P *value for trend test.

^b^Distant metastases or death within 5 years.

^c^Subset of the Stockholm cohort: 82 patients with tamoxifen 2–5 years, eight patients with tamoxifen <2 years, nine patients with tamoxifen in sequence with megesterolacetat, and five patients with tamoxifen and goserelin.

^d^One patient was removed because of missing data. The 5-year event was distant metastases within 5 years.

**Table 5:** Main biological functions among the present 64-gene set and the 70-gene set from the van't Veer study

Biological function	64-gene set (present study)	70-gene set (van't Veer and colleagues)
DNA replication/transcription/nucleosome assembly	IGF1, RRM2×2, TOP2A	ORC6L, MCM6, RFC4
	PFS2, CENPF, HOXA5, CBFA2T1, SETBP1, UHRF1, MKI67, HIST1H2BG, H2AFZ	KIAA1442, CENPA
Cell cycle/proliferation/growth	CDKN1C, PTN, CDC2×2,	EXT1, HEC, PRC1, NUSAP1 (LOC51203), MCM6, Cyclin E2, TGFB3, FLT1, FGF18, ECT2, GMPS, IGFBP5 × 2, ESM1, WISP1
	BUB1B, ESPL1, PRC1, TOP2A, NUSAP1 (LOC51203), CENPF, IGF1, RHOJ, UHRF1,PDGFD, CBFA2T1, NOV	
Apoptosis	ESPL1	FLT1
Cell adhesion/motility/invasion/metastasis	SLIT2, COL14A1, FLRT2, MFAP4, CLDN7, IGF1, RHOJ	WISP1, FGF18, MMP9
Angiogenesis	PTN	FLT1
Signal transduction	IGF1, RHOJ,	EXT1, GNAZ, GFBP5, CFFM4, FGF18, NMU, RAB6B, PK428, PK428 MP1, WISP1, DKFZP564D0462 (GPR126)
	PTN, DNM1, GPR56	
