# Supplementary material for: Gene expression profiling spares early breast cancer patients from adjuvant therapy: derived and validated in two population-based cohorts
Source: Breast Cancer Res. 2005 Oct 3;7(6):R953–64. doi: 10.1186/bcr1325 (PMC1410752; doi:10.1186/bcr1325)
Supplement: Additional file 1 — Supplementary Report presenting (i) details of gene filtering, (ii) details of cross-validation procedure to choose 64-gene signature, (iii) list of 64 genes, and (iv) other statistical analyses based on secondary endpoints. [file bcr1325-S1.doc]

# Supplementary Report 18 August 2005

**GENE EXPRESSION PROFILING SPARES EARLY BREAST CANCER PATIENTS FROM ADJUVANT THERAPY – DERIVED AND VALIDATED IN TWO POPULATION BASED COHORTS**

**Pawitan et al**

**Gene filter**: We started with 22,283 probe sets from U133A and 22,645 from U133B, and excluded all Affymetrix control genes (68 from each chip) and 100 housekeeping genes from U133B. This left us with 44,792 probe sets. We then included only genes that satisfy the following:

- present (P call by Affymetrix Expression analysis software) in more than 10% of the 159 patients. This gives 14,687 genes from U133A, and 11,041 genes from U133B, with a total of 25,728 genes.
- Showing sufficient biological variability across 159 patients, such that the 15^th^ smallest and the 15^th^ largest values have a minimum absolute difference of 1000 and a minimum fold difference of 3. This is a reasonable requirement if a gene were to be a useful biological marker. *The final numbers of genes included are 3393 from U133A and 3180 from U133B, for a total of 6573 genes.*

**Prediction method:** The diagonal linear discriminant analysis (LDA) (Dudoit, et al 2002). The genes are first ordered according to the standard two-sample t-tests, and they are entered into the list of genes used for class prediction based on their ranking. Equal number of genes from the top and the bottom of the list are included for prediction.

Class prediction using *k* genes was done using a diagonal linear discriminant analysis method (Dudoit et al, 2002), which is a variant of the standard maximum likelihood discrimination rule. Suppose x is a vector of the (log-) gene expression value from a tumor to be classified, and x_g_  is the expression value of gene *g*, and m_1g_ and m_0g_ are the means of the bad and good prognosis groups from the training set, and v_g_ is the variance, and a_g_ = (m_1g_ - m_0g_)/v_g_, and b_g_ = (m_1g_ + m_0g_)/2. The class predictor score is given by

S = sum_g_ a_g_ (x_g_ – b_g_),

where the summation is over the *k* selected genes. A patient with S>0 is assigned to the bad prognosis group, and otherwise to the good prognosis group. Thus, we will refer to S as the bad prognostic score.

**Full cross-validation using leave-one-out method:**

1. Remove one case for validation
2. Order the genes using two-sample t-test, and develop a class prediction using the rest of the samples (n=158 = 159-1)
3. Compute the bad prognosis score for the removed cases and predict using *k* genes. (This cross-validated bad prognosis score will be used also for multivariate analysis later.)
4. Repeat the procedure by removing each case in turn
5. Summarize the prediction performance by computing the error rate on the accumulated validation sample.

To choose the optimal number of genes, this procedure is repeated for *k* between 20 and 100. The plots below are based on the linear discriminant analysis; the cross-validated error rate is given on the y-axis and it is computed as a function of *k*. To get the minimal error rate on the bad prognosis score, which is equivalent to maximizing sensitivity in the group that might benefit from further therapy, we choose the optimal choice of *k*=64. The overall cross-validated error rate is around 33% (53/159), consisting of 36% (43/121) in the good prognosis group and 26% (10/38) in the bad prognosis group. Prediction of breast cancer events (deaths due to breast cancer and distant metastasis) is slightly better. Using the same class prediction equation, by applying it to the breast cancer events only, the error rate is reduced to 31%, consisting of 35% (45/128) in the good prognosis group and 16% (5/31) in the bad prognosis group.

**Table 1S.** Cross-validated prediction (Stockholm cohort)

All events

predicted

status good bad

good 78(64%) 43(36%)

bad 10 (26%) 28(74%)

Breast cancer events

predicted

status good bad

good 83(65%) 45(35%)

bad 5 (16%) 26(84%)

**Prediction on the training set.** 112 out of 159 cases (70%) were classified correctly. A total of 40 (33%) out of 121 patients with good prognosis, and 7 (18%) out of 38 patients with bad prognosis were wrongly classified. For breast cancer events, the total error rate is 30% (48/159), consisting of 34% (44/128) in the good prognosis group and 12% (4/31) in the bad prognosis group.

Analysis including clinical information

**Univariate comparison of clinical variables** We first compare the clinical characteristics of all the patients with good versus bad prognosis. This is first done using all deaths or distant relapse by five years as the clinical endpoint. Bad prognosis is associated with larger tumour size, PGR negative and lack of endocrine therapy.

**Table 3S.**

**Good (n=121) Bad (n=38) p-value**

Bad prognosis score 0.36 0.74 <0.0001

Age 57.5 (±12.4) 58.8 (±16.8) 0.59

Size (mm) 21.3 (±11.5) 25.6 (±12.6) 0.05

Size<21mm 0.65 0.47 0.06

Lymph 0.37 0.39 0.71

Grade 1 0.23 0.08 0.06 (combined test)

2 0.41 0.36

3 0.36 0.56

ER 0.83 0.79 0.61

PGR 0.77 0.55 0.01

Chemotherapy 0.18 0.21 0.69

Endocrine therapy 0.76 0.58 0.03

Radiotherapy 0.51 0.39 0.21

A similar comparison was also done by limiting the endpoint to distant relapse or deaths due to breast cancer.

**Table 4S.**

**Good (n=128) Bad (n=31) p-value**

Bad prognosis score 0.36 0.84 <0.0001

Age 58.5 (±12.9) 54.9 (±15.9) 0.19

Size 21.7 (±11.5) 25.1 (±13.4) 0.16

Size<21 0.63 0.52 0.30

Lymph 0.38 0.39 0.90

Grade 1 0.23 0.03 0.01 (combined test)

2 0.41 0.34

3 0.36 0.62

ER 0.83 0.77 0.49

PGR 0.76 0.55 0.02

Chemotherapy 0.17 0.26 0.27

Endocrine therapy 0.77 0.52 0.01

Radiotherapy 0.48 0.48 1.00

**Multivariate analysis:** From the training data we obtain the cross-validated bad prognosis score and use it in a multivariate analysis, by including standard clinical predictors such as age, stage, histologic grading, ER and PGR receptor status. To avoid biased estimates, the scores for patients in the training set were computed from the leave-one-out procedure, i.e. the score for a patient was computed by first removing the patient prior to computing the coefficients a_g_ and b_g_ from the optimal set of genes. The scores for patients in the testing set were computed using the full training set to compute the class predictor. Note, however, although these scores produce unbiased estimates, the standard error is likely to be optimistic because of dependence between the cross-validated values.

**Table 5S.**

**All endpoints (n=159, number of events =38)**

Odds-ratio (95% CI) P-value

Bad prognosis score 4.19 (1.49-11.77) 0.007

Age (per 10 years) 1.11 (0.79-1.54) 0.55

Stage

Stage 2 vs 1 1.28 (0.4-4.08) 0.68

Stage 3 vs 1 1.11 (0.42-2.95) 0.83

Elston grade

Grade 2 vs 1 3.32 (0.63-17.56) 0.16

Grade 3 vs 1 2.81 (0.5-15.74) 0.24

ER positive 2.94 (0.76-11.28) 0.12

PGR positive 0.35 (0.12-0.99) 0.05

**Table 6S.**

**Breast cancer endpoints (n=159, number of events= 31)**

Odds-ratio (95% CI) P-value

Bad prognosis score 10.64 (2.91-38.87) 0.0004

Age (per 10 years) 0.78 (0.53-1.14) 0.2

Stage

Stage 2 vs 1 1.6 (0.45-5.69) 0.47

Stage 3 vs 1 0.89 (0.3-2.69) 0.84

Eslton grade

Grade 2 vs 1 5.88 (0.6-57.25) 0.13

Grade 3 vs 1 3.11 (0.32-29.95) 0.33

ER positive 3.44 (0.78-15.21) 0.1

PGR positive 0.4 (0.13-1.28) 0.13

Survival analysis

As a comparison we also analysed the same data uses the full survival information, rather than simply the disease status at 5 years. The average followup time was 6.1 years, and the minimum followup for those who were censored was 5.6 years. There was an additional 8 events after 5 years, so the total number of events was 46. When deaths were limited to those due to breast cancer, the total number of events was 35. The Kaplan-Meier plot below shows a clear separation between the groups with good and bad prognosis scores.

**Cox regression** allows a multivariate analysis including the standard clinical variables in the model. The results are qualitatively similar as the logistic regression analysis of 5-year status.

**Table 7S.**

**All endpoints (n=159, number of events = 46)**

Hazard-ratio(95% CI) p-value

Bad prognosis score 3.53 (1.58-7.89) 0.002

Age (per 10 years) 1.1 (0.83-1.46) 0.49

Stage

Stage 2 vs 1 1.14 (0.45-2.86) 0.79

Stage 3 vs 1 1.28 (0.6-2.7) 0.52

Elston grade

Grade 2 vs 1 2.34 (0.66-8.3) 0.19

Grade 3 vs 1 1.65 (0.45-6.15) 0.45

ER positive 2.23 (0.84-5.91) 0.11

PGR positive 0.39 (0.18-0.83) 0.01

**Table 8S.**

**Breast cancer endpoints (n=159, number of events = 35)**

Hazard-ratio(95% CI) P-value

Bad prognosis score 6.73 (2.58-17.56) 0.0001

Age (per 10 years) 0.83 (0.6-1.14) 0.25

Stage

Stage 2 vs 1 1.26 (0.46-3.41) 0.65

Stage 3 vs 1 1.07 (0.46-2.49) 0.87

Elston grade

Grade 2 vs 1 3.01 (0.65-13.87) 0.16

Grade 3 vs 1 1.47 (0.31-7.03) 0.63

ER positive 2.36 (0.79-7.07) 0.12

PGR positive 0.47 (0.2-1.09) 0.08

| List of 64 genes. Genes with negative statistics are upregulated in the good prognosis group (good genes), and vice versa for genes with positive statistics. FDR = False discovery rate | | | |
| --- | --- | --- | --- |
| Number | Statistic (1-FDR) | Locus | Name |
| 1 | -5.49(1) | [---](http://www.ncbi.nlm.nih.gov/LocusLink/LocRpt.cgi?l=---) | ESTs |
| 2 | -5.26(1) | [80310](http://www.ncbi.nlm.nih.gov/LocusLink/LocRpt.cgi?l=80310) | spinal cord-derived growth factor-B |
| 3 | -5(1) | [1028](http://www.ncbi.nlm.nih.gov/LocusLink/LocRpt.cgi?l=1028) | cyclin-dependent kinase inhibitor 1C (p57, Kip2) |
| 4 | -4.49(1) | [3479](http://www.ncbi.nlm.nih.gov/LocusLink/LocRpt.cgi?l=3479) | insulin-like growth factor 1 (somatomedin C) |
| 5 | -4.47(1) | [---](http://www.ncbi.nlm.nih.gov/LocusLink/LocRpt.cgi?l=---) | ESTs |
| 6 | -4.4(1) | [3202](http://www.ncbi.nlm.nih.gov/LocusLink/LocRpt.cgi?l=3202) | homeo box A5 |
| 7 | -4.38(1) | [---](http://www.ncbi.nlm.nih.gov/LocusLink/LocRpt.cgi?l=---) | Homo sapiens, clone IMAGE:4246029, mRNA |
| 8 | -4.23(1) | [57722](http://www.ncbi.nlm.nih.gov/LocusLink/LocRpt.cgi?l=57722) | likely ortholog of mouse neighbor of Punc E11 |
| 9 | -4.21(1) | [219654](http://www.ncbi.nlm.nih.gov/LocusLink/LocRpt.cgi?l=219654) | hypothetical protein FLJ90798 |
| 10 | -4.2(1) | [9353](http://www.ncbi.nlm.nih.gov/LocusLink/LocRpt.cgi?l=9353) | slit homolog 2 (Drosophila) |
| 11 | -4.17(1) | [57381](http://www.ncbi.nlm.nih.gov/LocusLink/LocRpt.cgi?l=57381) | ras homolog gene family, member J |
| 12 | -4.17(1) | [79686](http://www.ncbi.nlm.nih.gov/LocusLink/LocRpt.cgi?l=79686) | chromosome 14 open reading frame 139 |
| 13 | -4.16(1) | [5764](http://www.ncbi.nlm.nih.gov/LocusLink/LocRpt.cgi?l=5764) | pleiotrophin (heparin binding growth factor 8, neurite growth-promoting factor 1) |
| 14 | -4.15(1) | [5348](http://www.ncbi.nlm.nih.gov/LocusLink/LocRpt.cgi?l=5348) | FXYD domain containing ion transport regulator 1 (phospholemman) |
| 15 | -4.15(1) | [7373](http://www.ncbi.nlm.nih.gov/LocusLink/LocRpt.cgi?l=7373) | collagen, type XIV, alpha 1 (undulin) |
| 16 | -4.12(1) | [---](http://www.ncbi.nlm.nih.gov/LocusLink/LocRpt.cgi?l=---) | Homo sapiens, clone IMAGE:5294728, mRNA |
| 17 | -4.06(1) | [---](http://www.ncbi.nlm.nih.gov/LocusLink/LocRpt.cgi?l=---) | Homo sapiens mRNA; cDNA DKFZp586N0121 (from clone DKFZp586N0121) |
| 18 | -4.05(1) | [6812](http://www.ncbi.nlm.nih.gov/LocusLink/LocRpt.cgi?l=6812) | syntaxin binding protein 1 |
| 19 | -4.05(1) | [10186](http://www.ncbi.nlm.nih.gov/LocusLink/LocRpt.cgi?l=10186) | lipoma HMGIC fusion partner |
| 20 | -4.01(1) | [6332](http://www.ncbi.nlm.nih.gov/LocusLink/LocRpt.cgi?l=6332) | sodium channel, voltage-gated, type VII, alpha polypeptide |
| 21 | -4(1) | [2205](http://www.ncbi.nlm.nih.gov/LocusLink/LocRpt.cgi?l=2205) | Fc fragment of IgE, high affinity I, receptor for; alpha polypeptide |
| 22 | -3.96(1) | [131583](http://www.ncbi.nlm.nih.gov/LocusLink/LocRpt.cgi?l=131583) | hypothetical protein FLJ90022 |
| 23 | -3.94(1) | [3479](http://www.ncbi.nlm.nih.gov/LocusLink/LocRpt.cgi?l=3479) | insulin-like growth factor 1 (somatomedin C) |
| 24 | -3.88(1) | [862](http://www.ncbi.nlm.nih.gov/LocusLink/LocRpt.cgi?l=862) | core-binding factor, runt domain, alpha subunit 2; translocated to, 1; cyclin D-related |
| 25 | -3.88(1) | [---](http://www.ncbi.nlm.nih.gov/LocusLink/LocRpt.cgi?l=---) | Homo sapiens mRNA; cDNA DKFZp586B211 (from clone DKFZp586B211) |
| 26 | -3.88(1) | [3479](http://www.ncbi.nlm.nih.gov/LocusLink/LocRpt.cgi?l=3479) | insulin-like growth factor 1 (somatomedin C) |
| 27 | -3.88(1) | [1759](http://www.ncbi.nlm.nih.gov/LocusLink/LocRpt.cgi?l=1759) | dynamin 1 |
| 28 | -3.86(1) | [8404](http://www.ncbi.nlm.nih.gov/LocusLink/LocRpt.cgi?l=8404) | SPARC-like 1 (mast9, hevin) |
| 29 | -3.85(1) | [4856](http://www.ncbi.nlm.nih.gov/LocusLink/LocRpt.cgi?l=4856) | nephroblastoma overexpressed gene |
| 30 | -3.84(1) | [26040](http://www.ncbi.nlm.nih.gov/LocusLink/LocRpt.cgi?l=26040) | SET binding protein 1 |
| 31 | -3.83(1) | [23768](http://www.ncbi.nlm.nih.gov/LocusLink/LocRpt.cgi?l=23768) | fibronectin leucine rich transmembrane protein 2 |
| 32 | -3.83(1) | [4239](http://www.ncbi.nlm.nih.gov/LocusLink/LocRpt.cgi?l=4239) | microfibrillar-associated protein 4 |
| 33 | 4.33(1) | [6183](http://www.ncbi.nlm.nih.gov/LocusLink/LocRpt.cgi?l=6183) | mitochondrial ribosomal protein S12 |
| 34 | 4.35(1) | [57510](http://www.ncbi.nlm.nih.gov/LocusLink/LocRpt.cgi?l=57510) | exportin 5 |
| 35 | 4.36(1) | [7153](http://www.ncbi.nlm.nih.gov/LocusLink/LocRpt.cgi?l=7153) | topoisomerase (DNA) II alpha 170kDa |
| 36 | 4.36(1) | [54443](http://www.ncbi.nlm.nih.gov/LocusLink/LocRpt.cgi?l=54443) | anillin, actin binding protein (scraps homolog, Drosophila) |
| 37 | 4.36(1) | [983](http://www.ncbi.nlm.nih.gov/LocusLink/LocRpt.cgi?l=983) | cell division cycle 2, G1 to S and G2 to M |
| 38 | 4.41(1) | [701](http://www.ncbi.nlm.nih.gov/LocusLink/LocRpt.cgi?l=701) | BUB1 budding uninhibited by benzimidazoles 1 homolog beta (yeast) |
| 39 | 4.44(1) | [6241](http://www.ncbi.nlm.nih.gov/LocusLink/LocRpt.cgi?l=6241) | ribonucleotide reductase M2 polypeptide |
| 40 | 4.44(1) | [51514](http://www.ncbi.nlm.nih.gov/LocusLink/LocRpt.cgi?l=51514) | RA-regulated nuclear matrix-associated protein |
| 41 | 4.44(1) | [1366](http://www.ncbi.nlm.nih.gov/LocusLink/LocRpt.cgi?l=1366) | claudin 7 |
| 42 | 4.45(1) | [10440](http://www.ncbi.nlm.nih.gov/LocusLink/LocRpt.cgi?l=10440) | translocase of inner mitochondrial membrane 17 homolog A (yeast) |
| 43 | 4.5(1) | [8339](http://www.ncbi.nlm.nih.gov/LocusLink/LocRpt.cgi?l=8339) | histone 1, H2bg |
| 44 | 4.51(1) | [9700](http://www.ncbi.nlm.nih.gov/LocusLink/LocRpt.cgi?l=9700) | extra spindle poles like 1 (S. cerevisiae) |
| 45 | 4.52(1) | [9055](http://www.ncbi.nlm.nih.gov/LocusLink/LocRpt.cgi?l=9055) | protein regulator of cytokinesis 1 |
| 46 | 4.58(1) | [10112](http://www.ncbi.nlm.nih.gov/LocusLink/LocRpt.cgi?l=10112) | kinesin family member 20A |
| 47 | 4.6(1) | [55165](http://www.ncbi.nlm.nih.gov/LocusLink/LocRpt.cgi?l=55165) | chromosome 10 open reading frame 3 |
| 48 | 4.61(1) | [983](http://www.ncbi.nlm.nih.gov/LocusLink/LocRpt.cgi?l=983) | cell division cycle 2, G1 to S and G2 to M |
| 49 | 4.68(1) | [195828](http://www.ncbi.nlm.nih.gov/LocusLink/LocRpt.cgi?l=195828) | zinc finger protein 367 |
| 50 | 4.7(1) | [29128](http://www.ncbi.nlm.nih.gov/LocusLink/LocRpt.cgi?l=29128) | ubiquitin-like, containing PHD and RING finger domains, 1 |
| 51 | 4.74(1) | [---](http://www.ncbi.nlm.nih.gov/LocusLink/LocRpt.cgi?l=---) | ESTs |
| 52 | 4.74(1) | [51203](http://www.ncbi.nlm.nih.gov/LocusLink/LocRpt.cgi?l=51203) | nucleolar protein ANKT |
| 53 | 4.8(1) | [3015](http://www.ncbi.nlm.nih.gov/LocusLink/LocRpt.cgi?l=3015) | H2A histone family, member Z |
| 54 | 4.83(1) | [259266](http://www.ncbi.nlm.nih.gov/LocusLink/LocRpt.cgi?l=259266) | asp (abnormal spindle)-like, microcephaly associated (Drosophila) |
| 55 | 4.87(1) | [79682](http://www.ncbi.nlm.nih.gov/LocusLink/LocRpt.cgi?l=79682) | hypothetical protein FLJ23468 |
| 56 | 4.9(1) | [51659](http://www.ncbi.nlm.nih.gov/LocusLink/LocRpt.cgi?l=51659) | HSPC037 protein |
| 57 | 4.98(1) | [991](http://www.ncbi.nlm.nih.gov/LocusLink/LocRpt.cgi?l=991) | CDC20 cell division cycle 20 homolog (S. cerevisiae) |
| 58 | 4.99(1) | [6241](http://www.ncbi.nlm.nih.gov/LocusLink/LocRpt.cgi?l=6241) | ribonucleotide reductase M2 polypeptide |
| 59 | 5(1) | [9768](http://www.ncbi.nlm.nih.gov/LocusLink/LocRpt.cgi?l=9768) | KIAA0101 gene product |
| 60 | 5.17(1) | [29089](http://www.ncbi.nlm.nih.gov/LocusLink/LocRpt.cgi?l=29089) | HSPC150 protein similar to ubiquitin-conjugating enzyme |
| 61 | 5.17(1) | [9289](http://www.ncbi.nlm.nih.gov/LocusLink/LocRpt.cgi?l=9289) | G protein-coupled receptor 56 |
| 62 | 5.18(1) | [4288](http://www.ncbi.nlm.nih.gov/LocusLink/LocRpt.cgi?l=4288) | antigen identified by monoclonal antibody Ki-67 |
| 63 | 5.4(1) | [1063](http://www.ncbi.nlm.nih.gov/LocusLink/LocRpt.cgi?l=1063) | centromere protein F, 350/400ka (mitosin) |
| 64 | 5.7(1) | [---](http://www.ncbi.nlm.nih.gov/LocusLink/LocRpt.cgi?l=---) | Homo sapiens, clone IMAGE:4826963, mRNA |

Classification of the 64 genes different biological functions according to the Gene Ontology.

| **Biological function** | **64 genes** | **70 genes** |
| --- | --- | --- |
| DNA replication | IGF1  RRM2 x 2  TOP2A  Pfs2  CENPF | ORC6L  MCM6  RFC4 |
| DNA transcription | HOXA5 (regulation)  CBFA2T1 (reg)  SETBP1 (reg)  TOP2A  UHRF1  MKI67 (reg) | MCM6  KIAA1442 |
| Nucleosome assembly | HIST1H2BG  H2AFZ | CENPA |
| Cell cycle | CDKN1C (neg reg)  PTN  CDC2 x2  BUB1B  ESPL1  PRC1  TOP2A  NUSAP1 (LOC51203)  CENPF | EXT1 (neg reg)  HEC  PRC1  NUSAP1 (LOC51203)  MCM6  Cyclin E2  TGFB3 |
| Cell proliferation | IGF1 (pos reg)  RHOJ  PTN (pos reg)  UHRF1 | FLT1 (pos reg)  TGFB3  FGF18 |
| Cell motility | IGF1  RHOJ |  |
| Chemotaxis | SLIT2 (induction of neg c) |  |
| Protein biosynthesis | MRPS12 |  |
| Protein ubiquitination | UHRF1 (ubiq protein-ligas)  HSPC150 (ubiqconjug enz) | UCH37 (ub thiolesterase) |
| Protein mitochondrial targeting | TIMM17A |  |
| Development | HOXA5 |  |
| Apoptosis | ESPL1 | BBC3 (PUMA) |
| Cell growth and/or maintenance | PDGFD  CBFA2T1  NOV (reg) | EXT1  ECT2  GMPS  IGFBP5 x 2  ESM1  TGFB3  WISP1 |
| Angiogenesis | PTN | FLT1 |
| Cell adhesion | SLIT2 (and Ca bind etc)  COL14A1  FLRT2  MFAP4  CLDN7 (tight junction) | WISP1 |
| Invasion |  | FGF18 |
| Cell-cell signaling |  | WISP1  TGFB3  FGF18 |
| Metastasis | PTN | MMP9 |
| Extracellular matrix organization and biogenesis |  | COL4A2 |
| Collagen catabolism |  | MMP9 |
| Receptor signalling | FLRT2 |  |
| Signal transduction |  | EXT1 (not specified, wnt?)  GNAZ (G protein coupled)  IGFBP5 (not specified)  CFFM4  FGF18  NMU |
| Ras protein signal transduction | IGF1 x 3 |  |
| Small GTPase mediated signal transduction | RHOJ | RAB6B  PK428 |
| Transmembrane receptor protein tyrosine phosphatase signal transduction | PTN |  |
| MAPK cascade |  | MP1 |
| Wnt receptor signaling pathway |  | WISP1 |
| Receptor mediated endocytosis | DNM1 |  |
| Neuropeptide signaling pathway | GPR56 | DKFZP564D0462 (GPR126) |
| Metabolism | GPR56 | OXCT (succinyl-CoA)  FLJ12443  DCK (pyrimidine metabolism)  SM-20 (protein metab)  SLC2A3 (carbohydrate)  FLJ11354 (DNA restriction)  PECI (fatty acid)  GSTM3  ALDH4 (alcohol, lipid) |
| Synaptic transmission | DNM1 |  |
| Ion transport | FXYD1 (chloride transport)  SCN7A (cation transport) |  |
| Protein transport | XPO5  KIF20A | KIAA 1067 (EXOC7)  RAB6B  SLC2A3  AP2B1 |
| Immune response | FCER1A |  |
| Energy pathways | CBFA2T1 |  |
| Unknown | 11  NOPE  STXBP1  LHFP  FAM43A  SPARCL1 (Ca binding)  ANLN  L2DTL (RAMP)  ZNF367  ASPM  MLF1IP  KIAA0101 | 16 (not annotated sequences hypothetical proteins)  DC13  SERF1A  L2DTL (RAMP)  KIAA 0175  AKAP2  TMEFF1  FLJ11190  FLJ22477  LOC57110  HSA250839  CEGP1  KIAA1442 |
